# Retrospective Phylogenetic Analysis of Mayaro Virus, French Guiana, 1996–2024

**DOI:** 10.3201/eid3205.251435

**Published:** 2026-05

**Authors:** Alisé Lagrave, Antoine Enfissi, Sourakhata Tirera, Loïc Epelboin, Jean-Bernard Duchemin, Tiphanie Succo, Anne Lavergne, Dominique Rousset

**Affiliations:** Institut Pasteur de la Guyane, Arbovirus National Reference Center, Virology Unit, Cayenne, French Guiana (A. Lagrave, A. Enfissi, S. Tirera, A. Lavergne, D. Rousset); CHU de Guyane, Unité des Maladies Infectieuses et Tropicales, Cayenne (L. Epelboin); CHU de Guyane, CIC Inserm, Santé des Populations Amazoniennes, Cayenne (L. Epelboin); Institut Pasteur de la Guyane, Unité Entomologie médicale, Cayenne (J.-B. Duchemin); Santé Publique France, Cellule Guyane, Cayenne (T. Succo)

**Keywords:** Mayaro virus, viruses, zoonoses, vector-borne infections, mosquitoes, phylogenetic analysis, French Guiana

## Abstract

We conducted a retrospective phylogenetic analysis of Mayaro virus (MAYV) detected in French Guiana during 1996–2024. Analysis revealed circulation of MAYV genotype D sublineage 2 and suggested introduction from Brazil and spread to Haiti and Venezuela. Phylogenetic findings support endemic circulation and reinforce the need for MAYV surveillance in the region.

Mayaro virus (MAYV), a mosquitoborne RNA virus of the genus *Alphavirus* (family Togaviridae), causes acute febrile illness, often accompanied by prolonged arthralgia (*1*). Identified in 1954 in Trinidad and Tobago, MAYV has caused sporadic outbreaks throughout Central and South America (*2*–*5*). Clinical manifestations of MAYV infection include fever, headache, myalgia, nausea, and persistent joint pain, sometimes lasting more than a year (*6*,*7*). 

Among arboviruses in the Amazon region, MAYV and emerging Oropouche virus are considered to have the highest epidemic potential (*1*,*2*). Phylogenetic analyses have identified 3 MAYV genotypes, D, L, and N (*8*–*10*). Genotype D is widely distributed, L is restricted to Brazil, and N has only been described from Peru, but evidence suggests recombination among MAYVs, as for other alphaviruses (*8*–*10*). 

MAYV is primarily maintained in a sylvatic cycle involving *Haemagogus janthinomys* mosquitoes and nonhuman primates, with occasional spillover to humans (*2*). However, experimental studies with urban vectors *Aedes aegypti* and *Ae. albopictus* mosquitoes have shown them to be competent MAYV vectors, raising concerns about urban emergence (*11*). 

In French Guiana, serologic studies support endemic sylvatic MAYV transmission, but an increase in urban and periurban cases in 2020 suggested a possible epidemiologic shift, reminiscent of adaptations observed in chikungunya virus (*7*,*12*,*13*). However, MAYV remains a neglected pathogen, and genomic data are scarce. By 2024, only 2 complete genomes were publicly available, and no comprehensive evolutionary analyses had been conducted for French Guiana. To address those gaps, we conducted a retrospective genomic analysis of virologically confirmed MAYV infections to explore potential genetic markers that could be associated with shifting transmission patterns and potential adaptation to new ecologic niches.

## The Study

In French Guiana, the National Reference Center for Arboviruses collects serum samples for diagnostic and surveillance purposes (Appendix). During 1996–2024, French Guiana reported 38 cases of MAYV infection, including 2 exported cases, 1 to Germany and 1 to mainland France, and 4 cases that seroconverted without PCR confirmation. Cases were sporadic during 1996–2019, especially during 2005–2016, when specific surveillance was lacking. In 2020, a cluster of 14 cases occurred within 3 months, mainly in Cayenne and surrounding areas, most without identified epidemiologic links. In 2024, four additional PCR-confirmed cases were detected, 2 linked to the Nouragues Nature Reserve and 2 from the western and central coastal regions.

Overall, French Guiana confirmed 34 infections by quantitative reverse-transcription PCR (qRT-PCR) or viral isolation in C6/36 cells; 32 were diagnosed locally (Appendix Figure). From those cases, we obtained 25 complete genomes, including 24 sequences we generated (Appendix Table 1). We performed whole-genome sequencing by using an amplicon-based MinION protocol (Oxford Nanopore Technologies, https://nanoporetech.com) and in-house primers (Appendix Table 2). We generated consensus genomes by using the ARTIC pipeline (ARTIC Network, https://artic.network) with Medaka polishing and completed missing regions by using Sanger sequencing (Appendix). 

For phylogenetic analyses, we retrieved 76 complete coding sequences from GenBank and combined those with the 24 newly generated genomes for a total of 100 sequences. Using the Recombination Detection Program 4 (https://web.cbio.uct.ac.za/~darren/rdp.html), we detected no recombination events among French Guiana strains. We used a dataset of 45 genotype D sublineage 2 sequences to refine evolutionary inferences. We reconstructed Bayesian time-scaled phylogenies under a general time-reversible plus gamma distribution plus invariable site model with a strict molecular clock and Bayesian skyline prior plots; all parameters showed adequate convergence (effective sample size >200).

**Figure 1:**
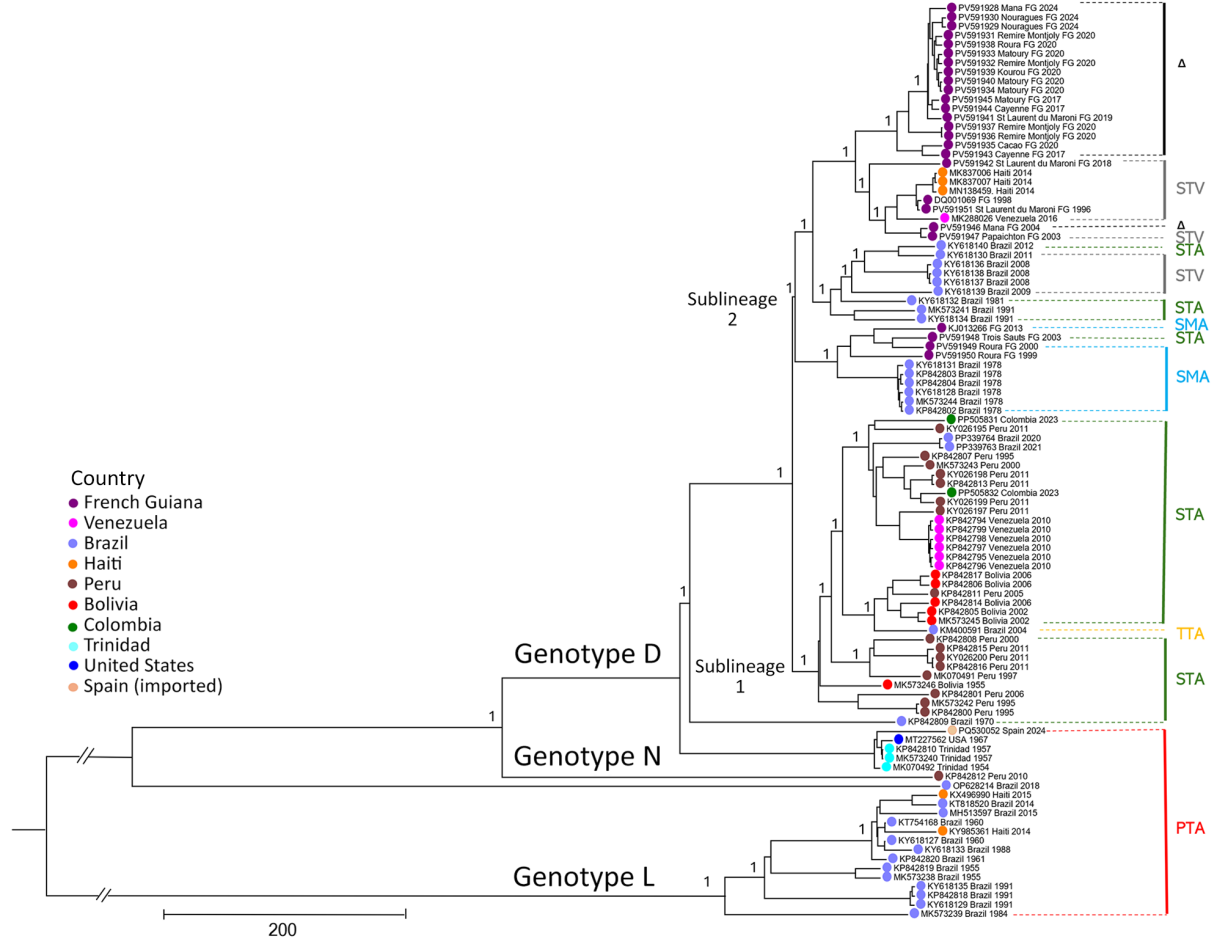
Bayesian phylogeny of 100 coding sequences from a retrospective phylogenetic analysis of Mayaro virus, French Guiana, 1996–2024. Maximum clade credibility tree inferred by using the general time-reversible with gamma distribution and invariant site substitution model, under a strict clock and Bayesian skyline coalescent prior. The tree was generated by using TreeAnnotator version 1.10.4 (BEAST Developers, https://beast.community/treeannotator), and the resulting time-scaled phylogenies were visualized with FigTree version 1.4.3 (https://tree.bio.ed.ac.uk/software/ﬁgtree). The tree includes major genotypes L, N, and D, and sublineages 1 and 2. The amino acid motifs at positions 1714–1716 are shown in color on the right side of the figure; triangles indicate strains with STV deletions. Bootstrap support values are indicated on the corresponding branches; a value of 1 corresponds to 100% bootstrap support. GenBank accession numbers are provided. Scale bar indicates nucleotide substitutions per site.

**Figure 2:**
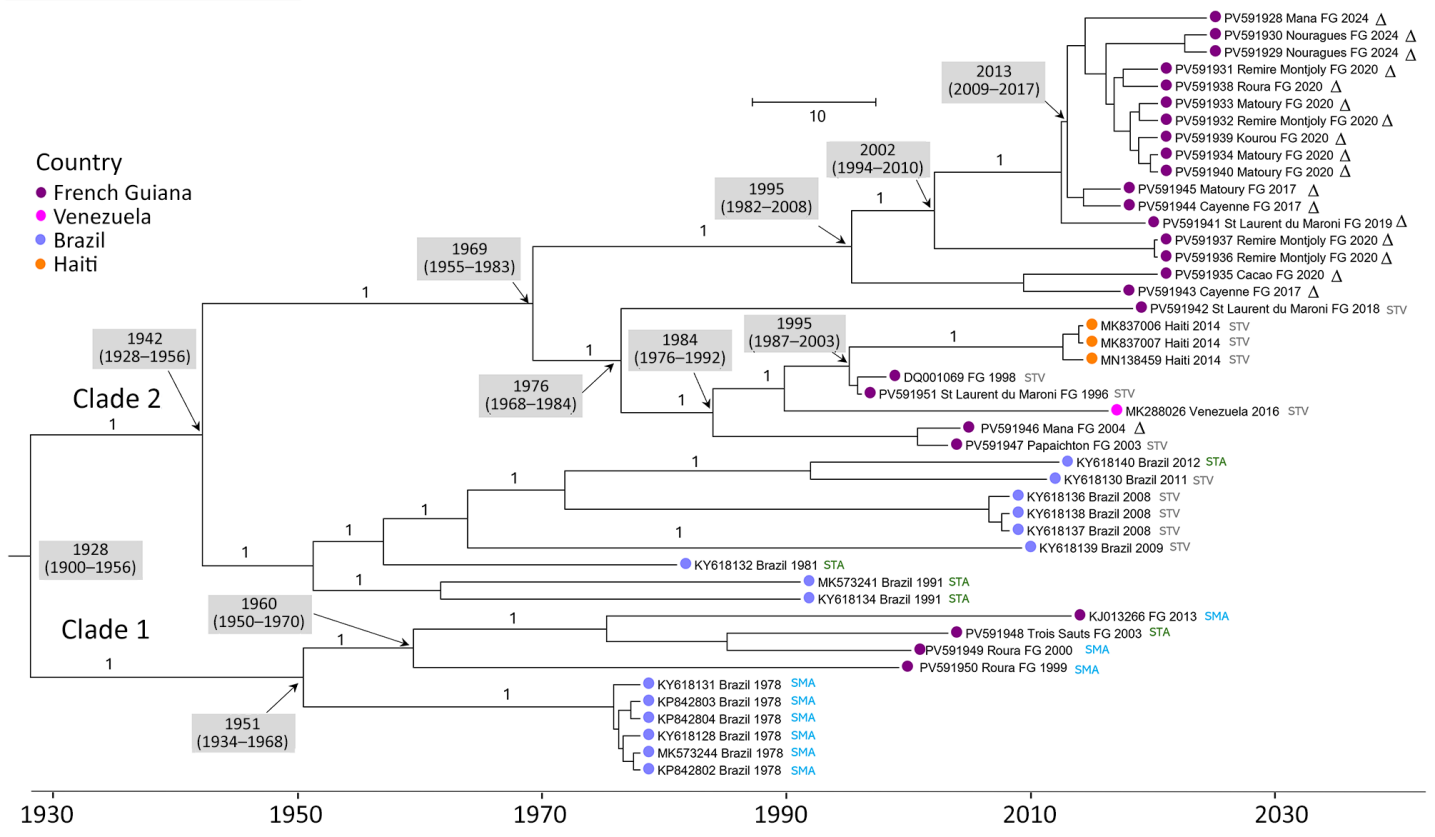
Bayesian phylogeny of 45 selected genotype D sublineage 2 coding sequences from a retrospective phylogenetic analysis of Mayaro virus, French Guiana, 1996–2024. Maximum clade credibility tree inferred by using the general time-reversible with gamma distribution and invariant site substitution model, under a strict clock and Bayesian skyline coalescent prior. The tree was generated by using TreeAnnotator version 1.10.4 (BEAST Developers, https://beast.community/treeannotator), and the resulting time-scaled phylogenies were visualized with FigTree version 1.4.3 (https://tree.bio.ed.ac.uk/software/ﬁgtree). Amino acid motifs at positions 1714–1716 are indicated by color at the terminal nodes of each sequence. Gray shaded boxes indicate dates of time to most recent common ancestor (95% highest posterior density). Bootstrap support values are indicated on the corresponding branches; a value of 1 corresponds to 100% bootstrap support. GenBank accession numbers are provided. Scale bar indicates nucleotide substitutions per site.

Our analyses showed that all French Guiana MAYV strains identified during 1996–2024 belonged to genotype D, and local strains shared high (97.58%–99.98%) nucleotide identity (Figure 1). Within global genotype D sequences, we identified 2 major sublineages: sublineage 1, which included sequences from Peru, Brazil, Bolivia, and Venezuela; and sublineage 2, which included sequences from French Guiana, Brazil, Haiti, and Venezuela. French Guiana sequences within sublineage 2 formed 2 clades that had a time to most recent common ancestor (tMRCA) of 1928 (95% highest posterior density [HPD] 1900–1956) (Figure 2).

Sublineage 2 clade 1 included sequences from Brazil from 1978 and French Guiana strains from 1999–2013 (tMRCA 1951, 95% HPD 1934–1968). Sublineage 2 clade 2 comprised sequences from Brazil from 1981–2012, and most French Guiana strains collected during 1996–2024; in addition, strains from Haiti from 2014 and from Venezuela from 2016 grouped within the French Guiana subclade. We estimated the sublineage 2 clade 2 tMRCA at 1942 (95% HPD 1928–1956). 

Amino acid analyses revealed variability in nonstructural protein 3 at positions 1714–1716. Of note, sublineage 1 predominantly exhibited an STA motif, but sublineage 2 showed greater diversity, including SMA, STV, and a deletion shared French Guiana strains since 2004, consistent with ongoing local diversification.

All strains circulating in French Guiana belonged exclusively to genotype D, consistent with its broad distribution in South and Central America (*3*–*5*,*8*,*14*). The presence of Brazil sequences in both clades supports historical introductions from Brazil followed by sustained local transmission. Conversely, clustering of sequences from Haiti and Venezuela within a French Guiana subclade suggests possible secondary exportations, although we cannot exclude sampling bias given the limited number of recent genomes available (*3*–*5*).

High nucleotide identity among French Guiana strains and across sublineage 2, combined with relatively recent estimates of tMRCA, support long-term endemic circulation with limited genetic divergence. Phylogeographic patterns further indicate progressive spatial expansion: early clade 1 strains were mainly confined to inland and eastern areas, whereas clade 2 strains spread from western regions eastward and toward urban coastal centers, including Cayenne and surrounding municipalities. That distribution is consistent with persistent local transmission within a relatively stable ecologic niche, punctuated by episodic emergence.

The unusual cluster of 14 urban and periurban cases in 2020 raised concerns about a potential epidemiologic shift from a predominantly sylvatic cycle toward increased urban transmission (*11*,*15*). However, we detected no recombination events among French Guiana genomes and did not identify any mutation specifically associated with the 2020 cases. Enhanced diagnostic efforts, particularly during the COVID-19 pandemic and concurrent dengue outbreaks, likely improved case detection and could partly explain the 2020 case increase.

Amino acid analyses revealed variability at positions 1714–1716 in nonstructural protein 3, including a recurrent STV deletion in clade 2 strains sharing a common ancestor around 1995. Of note, we observed that deletion in strains from both urban and remote forest areas, arguing against a clear association with ecologic shift or vector change. Thus, alternative explanations must be considered, including spillover enabled by increasing overlap between forest fragments and expanding urban areas or competence of urban vectors such as *Ae. aegypti* and *Ae. albopictus* mosquitoes (although absent in French Guiana), which experimental studies demonstrated as viable vectors (*11*,*13*,*15*). 

## Conclusions

Genomic studies of MAYV remain limited, reflecting the continued neglect of this virus despite its broad distribution in the Amazon Basin and outbreaks in South America and the Caribbean (*3*–*5*). Few complete genomes are publicly available, restricting robust phylogeographic analyses and assessment of emergence potential. This study provides insights into the long-term evolutionary dynamics of MAYV in French Guiana and increases the total number of publicly available sequences. However, historical surveillance gaps and underdiagnosis suggest that current genomic data underestimate MAYV diversity. 

Overall, our findings support longstanding endemic circulation of genotype D in French Guiana, characterized by geographic structuring and limited diversification, underscoring the need for integrated genomic, ecologic, and entomologic surveillance strategies. Expanded, sustained genomic surveillance across South America is essential to improving phylogenetic resolution, monitoring viral evolution, and assessing urbanization risk.

Appendix Additional information on a retrospective phylogenetic analysis of Mayaro virus, French Guiana, 1996–2024.

## References

[1] Caicedo EY, Charniga K, Rueda A, Dorigatti I, Mendez Y, Hamlet A, et al. The epidemiology of Mayaro virus in the Americas: a systematic review and key parameter estimates for outbreak modelling. PLoS Negl Trop Dis. 2021;15:e0009418. 10.1371/journal.pntd.0009418 34081717 PMC8205173

[2] Wei LLL, Tom R, Kim YC. Mayaro virus: an emerging alphavirus in the Americas. Viruses. 2024;16:1297. 10.3390/v16081297 39205271 PMC11359717

[3] Forato J, Meira CA, Claro IM, Amorim MR, de Souza GF, Muraro SP, et al. Molecular epidemiology of Mayaro virus among febrile patients, Roraima State, Brazil, 2018–2021. Emerg Infect Dis. 2024;30:1013–6. 10.3201/eid3005.231406 38666638 PMC11060474

[4] Halsey ES, Siles C, Guevara C, Vilcarromero S, Jhonston EJ, Ramal C, et al. Mayaro virus infection, Amazon Basin region, Peru, 2010–2013. Emerg Infect Dis. 2013;19:1839–42. 10.3201/eid1911.130777 24210165 PMC3837653

[5] Lednicky J, De Rochars VMB, Elbadry M, Loeb J, Telisma T, Chavannes S, et al. Mayaro virus in child with acute febrile illness, Haiti, 2015. Emerg Infect Dis. 2016;22:2000–2. 10.3201/eid2211.161015 27767924 PMC5088037

[6] Acosta-Ampudia Y, Monsalve DM, Rodríguez Y, Pacheco Y, Anaya JM, Ramírez-Santana C. Mayaro: an emerging viral threat? Emerg Microbes Infect. 2018;7:1–11. 10.1038/s41426-018-0163-5 30254258 PMC6156602

[7] Mutricy R, Matheus S, Mosnier É, Martinez-Lorenzi E, De Laval F, Nacher M, et al. Mayaro virus infection in French Guiana, a cross sectional study 2003–2019. Infect Genet Evol. 2022;99:105243. 10.1016/j.meegid.2022.105243 35151887

[8] Auguste AJ, Liria J, Forrester NL, Giambalvo D, Moncada M, Long KC, et al. Evolutionary and ecological characterization of Mayaro virus strains isolated during an outbreak, Venezuela, 2010. Emerg Infect Dis. 2015;21:1742–50. 10.3201/eid2110.141660 26401714 PMC4593426

[9] Mavian C, Rife BD, Dollar JJ, Cella E, Ciccozzi M, Prosperi MCF, et al. Emergence of recombinant Mayaro virus strains from the Amazon basin. Sci Rep. 2017;7:8718. 10.1038/s41598-017-07152-5 28821712 PMC5562835

[10] Marinho MDS, Ferreira GM, Grosche VR, Nicolau-Junior N, Campos TL, Santos IA, et al. Evolutionary profile of Mayaro virus in the Americas: an update into genome variability. Viruses. 2024;16:809. 10.3390/v16050809 38793690 PMC11126029

[11] Long KC, Ziegler SA, Thangamani S, Hausser NL, Kochel TJ, Higgs S, et al. Experimental transmission of Mayaro virus by *Aedes aegypti.* Am J Trop Med Hyg. 2011;85:750–7. 10.4269/ajtmh.2011.11-0359 21976583 PMC3183788

[12] Bonifay T, Le Turnier P, Epelboin Y, Carvalho L, De Thoisy B, Djossou F, et al. Review on main arboviruses circulating on French Guiana, an ultra-peripheric European region in South America. Viruses. 2023;15:1268. 10.3390/v15061268 37376570 PMC10302420

[13] Hozé N, Salje H, Rousset D, Fritzell C, Vanhomwegen J, Bailly S, et al. Reconstructing Mayaro virus circulation in French Guiana shows frequent spillovers. Nat Commun. 2020;11:2842. 10.1038/s41467-020-16516-x 32503971 PMC7275077

[14] Powers AM, Aguilar PV, Chandler LJ, Brault AC, Meakins TA, Watts D, et al. Genetic relationships among Mayaro and Una viruses suggest distinct patterns of transmission. Am J Trop Med Hyg. 2006;75:461–9. 10.4269/ajtmh.2006.75.461 16968922

[15] Mackay IM, Arden KE. Mayaro virus: a forest virus primed for a trip to the city? Microbes Infect. 2016;18:724–34. 10.1016/j.micinf.2016.10.007 27989728

