# Supplementary material for: Retrospective Phylogenetic Analysis of Mayaro Virus, French Guiana, 1996–2024
Source: Emerg Infect Dis. 2026 May;32(5):740–4. doi: 10.3201/eid3205.251435 (PMC13174963; doi:10.3201/eid3205.251435)
Supplement: Appendix — Additional information on a retrospective phylogenetic analysis of Mayaro virus, French Guiana, 1996–2024. [file 25-1435-Techapp-s1.pdf]

Article DOI: https://doi.org/10.3201/eid3205.251435

EID cannot ensure accessibility for supplementary materials supplied by authors. Readers who have difficulty accessing supplementary content should contact the authors for assistance.

Retrospective Phylogenetic Analysis of Mayaro Virus, French Guiana, 1996–2024

Appendix

Materials and Methods supplementary data

Ethical Statement

This study was carried out within the framework of the public health surveillance program led by NRCA-FG, in partnership with the national public health agency (Santé Publique France, SpF). As the research was part of routine epidemiologic surveillance, consultation with an ethics committee was not required. The human serum samples used in this study are part of the samples received at the NRCA-FG for diagnostic or surveillance purposes. These samples are stored in the NRCA-FG biobank in accordance with French legislation (Article L.1211–2 and related provisions of the French Public Health Code). All serum samples were anonymized, ensuring no or minimal risk to individuals, in compliance with the European General Data Protection Regulation and the guidelines of the French National Commission on Informatics and Liberty (CNIL).

Clinical Samples and Study Design

Samples used in this study were selected from human sera collected as part of the diagnostic and surveillance activities of the NRCA-FG. Between 1996 and 2024, 38 cases of MAYV infections were reported in French Guiana, including two exported cases identified in Germany and France, and four seroconversions without any PCR confirmation. Few sporadic cases have been detected between 1996 and 2019, except between 2005–2016 due to the lack of MAYV surveillance during this period. Surprisingly, in 2020, 14 cases were reported over a 3-month period, most of them from Cayenne and surroundings, with no epidemiologic links identified among them, except for one couple. In 2024, four PCR-confirmed cases were

identified: two in individuals returning from the Nouragues Nature Reserve in northeastern French Guiana, one in Saint-Laurent-du-Maroni and another in Cayenne. The locations of the different presumed sites of contamination are shown in Appendix Figure, Appendix Table 1.

A total of 34 MAYV infections were confirmed by reverse transcription qPCR (RT-qPCR) or viral isolation on C6/36 insect cell lines—32 at the NRCA-FG and two abroad. Of the 32 cases confirmed at NRCA-FG, 25 complete viral genome sequences were obtained, including 24 newly identified sequences (Appendix Table 1).

MinION Library Preparation and Multiplexed Nanopore Sequencing

Whole-genome sequencing was performed on a MinION device (Oxford Nanopore Technologies, Oxford, UK) based on a protocol from Quick et al., 2017. Briefly, RNAs were first extracted from serum or virus isolated in cell culture using the Macherey Nagel Virus 96 kit (Macherey Nagel, Düren, Germany; ref: 740452.4) and reverse transcribed to cDNA using LunaScript RT SuperMix (NEB, Ipswich, MA, USA) following the manufacturer's instructions. A polymerase chain reaction (PCR) was performed with Q5 High-Fidelity DNA polymerase (NEB Ipswich, MA, USA) with primer designed described in Appendix Table 2. The resulting PCR products were pooled, cleaned using AmpureXP magnetic beads (Beckman Coulter, High Wycombe, UK), and quantified using a Qubit dsDNA High Sensitivity assay on a Qubit 3.0 instrument (Thermo Fisher Scientific, Waltham, MA, USA). Sequencing libraries were loaded onto the R9.4 or R10.4 flow cells (Oxford Nanopore Technologies, Oxford, UK), and sequence reads were base-called and demultiplexed using the Guppy algorithm v3.6 (Oxford Nanopore Technologies, Oxford, UK).

Sanger sequencing

Amplicon dropouts were resolved by Sanger sequencing; both strands were sequenced and only sequences achieving a quality score greater than 75% were retained for incorporation into the final consensus. Sanger sequence traces were visualized and examined using CLC Main Workbench software (version 22; Qiagen, Hilden, Germany).

Bioinformatics analyses

Genome reconstruction was performed using the ARTIC Network field bioinformatics pipeline and the corresponding primer schemes (Appendix Table 2). Consensus genome sequences were generated with the ARTIC bioinformatics pipeline (version 1.2.4), using

reference genome MK837007 and the Medaka variant-calling algorithm implemented in Medaka.

Sequencing with R9.4.1 (R9.10) Flow Cells

Resulting FASTQ files were processed using the ARTIC pipeline (version 1.2.1). Reads were length-filtered with the guppyplex module to retain fragments within the expected amplicon size range and quality-filtered with a minimum Phred score of 7. Consensus sequences were generated using the nanopolish workflow within ARTIC, which performs variant calling based on raw nanopore signal data.

Sequencing with R10.4 Flow Cells

Raw POD5 signal files were basecalled in high-accuracy or super high-accuracy mode using either Guppy or Dorado. Reads were processed using the ARTIC pipeline (version 1.2.4) with guppyplex, applying a minimum quality threshold of 8. Consensus sequences were generated using the Medaka workflow in combination with the corresponding basecalling model.

For all analyses, default pipeline parameters were retained unless otherwise specified. A minimum depth of coverage of 20× was required for consensus calling, and primer trimming was performed within the ARTIC workflow. Intermediate BAM and VCF files were systematically inspected to validate consensus sequences and perform manual corrections when necessary.

Sequence Analysis and Phylogenetic Analysis

All complete MAYV sequences available on NCBI (n = 76, downloaded on 20 March 2025) were added to the 24 FG sequences generated in this study, forming the first dataset (n = 100 sequences). This dataset was trimmed to include only coding sequences (CDS). The study of recombination events was conducted on this dataset using Recombinaison Detection Program version 4 (RDP4) software. In addition, the nucleotide alignment of dataset 1 was translated and aligned at the protein level, and this amino acid alignment was manually examined using CLC Main Workbench software (version 22; Qiagen, Hilden, Germany) to investigate potential amino acid changes, insertions, or deletions.

To investigate the evolution of FG strains more precisely and estimate their MRCA, a second dataset was created. This dataset consisted solely of sequences belonging to Genotype D, sublineage 2, at the CDS level (n = 45 sequences).

Phylogenetic analyses were conducted using BEAST and BEAUTI (version 1.10.1), employing the General Time Reversible model with gamma distribution and invariant sites (GTR+G+I). This substitution model was identified as the best fit by CLC Main Workbench software (version 22; Qiagen, Hilden, Germany) and MEGA software based on the corrected Akaike information criterion (AICc). Both strict and relaxed molecular clock models were evaluated in BEAST; the strict molecular clock was ultimately selected because it demonstrated better statistical performance, with higher and more stable effective sample size (ESS) values and improved convergence diagnostics. A strict molecular clock and Bayesian Skyline coalescent prior were therefore applied in the final analyses. Markov chain Monte Carlo (MCMC) analyses were run for 100 million generations for the first dataset and 10 million generations for the second, with sampling every 1,000 generations. Convergence was assessed in Tracer v1.7.1, with the first 10% of samples discarded as burn-in. Effective sample sizes (ESS) exceeded 200 for all estimated parameters, indicating sufficient convergence. Maximum clade credibility (MCC) trees were generated using TreeAnnotator v1.10.4, and the resulting time-scaled phylogenies were visualized with FigTree v1.4.3 (http://tree.bio.ed.ac.uk/software/figtree/, accessed on 07 July 2025).

Appendix Table 1. Overview of 24 Newly Characterized MAYV Sequences from French Guiana Including GenBank Accession Numbers, Collection Dates, Presumed site of contamination, Isolation Sources (P1: passage 1 and P2: passage 2), and Sequence Lengths.

GenBank accession no.	Collection dates	Municipality and precise site if available	Isolation sources	Sequence length, bp
PV591928	2024–02–15	Mana (Saut Maman-Valentin)	Serum	11,337
PV591929	2024–02–01	Regina (Nouragues National Nature Reserve)	Serum	11,341
PV591930	2024–02–27	Regina (Nouragues National Nature Reserve)	C6/36 insect cell lines P1	11,359
PV591931	2020–07–22	Rémire-Montjoly	C6/36 insect cell lines P1	11,339
PV591932	2020–07–25	Rémire-Montjoly	C6/36 insect cell lines P1	11,339
PV591933	2020–07–31	Matoury	Serum	11,332
PV591934	2020–08–14	Matoury	C6/36 insect cell lines P2	11,325
PV591935	2020–08–03	Roura (Cacao)	C6/36 insect cell lines P1	11,340
PV591936	2020–09–01	Rémire-Montjoly	C6/36 insect cell lines P1	11,194
PV591937	2020–09–01	Rémire-Montjoly	Serum	11,194
PV591938	2020–08–18	Roura	C6/36 insect cell lines P1	11,339
PV591939	2020–08–17	Kourou	C6/36 insect cell lines P1	11,339
PV591940	2020–09–11	Matoury	C6/36 insect cell lines P2	11,339
PV591941	2019–07–26	Saint-Laurent-du-Maroni	Serum	11,339
PV591942	2018–10–09	Saint-Laurent-du-Maroni	Serum	11,313
PV591943	2017–08–01	Cayenne	C6/36 insect cell lines P1	11,347
PV591944	2017–08–26	Cayenne	C6/36 insect cell lines P1	11,319
PV591945	2017–05–03	Matoury	C6/36 insect cell lines P1	11,325
PV591946	2004–02–05	Mana	C6/36 insect cell lines P2	11,341
PV591947	2003–09–26	Papaïchton	C6/36 insect cell lines P2	11,350
PV591948	2003–05–30	Camopi (Trois Sauts)	C6/36 insect cell lines P2	11,349
PV591949	2000–04–12	Roura (Kaw)	C6/36 insect cell lines P2	11,349
PV591950	1999–08–03	Roura (Nancibo)	C6/36 insect cell lines P2	11,302
PV591951	1996–02–29	Saint-Laurent-du-Maroni (Chutes Voltaire)	C6/36 insect cell lines	11,349

Appendix Table 2. Primers for complete genome MAYV amplification.

Name	Sequences (5′→3′)	Position KX496990
May1F	TTA GAG ACG ACC ATG GCG G	1–19
May1Fbis	CGG GCA AGT GAC ACT TGT TC	17–36
May1R	CGT AGC CCT CAC ATG ACA C	926–944
May2F	TGA AAG CCA GGA ACA TAG GA	702–721
May2R	TCG ACA ACA CCG GCT CC	1691–1707
May3F	GCC AGA GCA GCA GAG A	1553–1568
May3R	GTC GYA CCG TGG CGA TAA G	2489–2507
May4F	GGA CTG AAA CGA AGA CCR G	2198–2216
May4R	TCG TTG ACC TTC ATT CGG AC	2873–2892
May5F	GTT GAC CCG AAA GGG CGT	2848–2865
May5R	GCT CTC AAA AGG AGG CTT CC	3851–3870
May6F	TCG TCA ACA TGC AYA CAC CG	3741–3760
May6R	TGC TCC CAC GTT TTA TCG CG	4520–4539
May7F	CGT CAA GTG CGA CGA GAC	4261–4278
May7R	CTT GTG ACT CTC TCY GGT GTC	1894–4914
May8F	GTA TGC RCT GGG CGA GA	4786–4802
May8R	CGA TCC ATA ATC GGC ACC TC	5648–5667
May9F	TCT CAT CMG ACG TCG GTC C	5589–5607
May9R	GCA GAC ATG TCA AAC AGG GT	6611–6630
May10F	GTG ATT CAA GCT GCC GAG C	6512–6530
May10R	CCT ATT TAG GAC CGC CGT AC	7399–7418
May11F	GAT GAA GAC CGC CGT AGG	7208–7225
May11R	CTG TCG CCA GGC TTA CC	8056–8072
May12F	GTG GCT ATG AAG TCA GAT GC	7939–7958
May12R	AAT GTC CGG CGG CAT GT	8924–8940
May13F	ACC GCC GAA ACC TCT GAA G	8893–8911
May13R	GCA ACA TGA GCC TTA TAC GGG	9900–9920
May14F	CGT TGC CAG TGC TTA CGA G	9849–9867
May14R	CGG TGA GTC CCT GTC TT	10595–10611
May15F	CGG GCA GCC TGG AAG AT	10443–10459
May15R	GGT TAG TAA GTG CCC TAT AGT GGC	11407–11430
May15Rbis	TAG GTG GTT ATT ATA TGC GCC	11360–11380
May15Rter	CCA GCC CGC ATT ATA CAC G	11184–11202

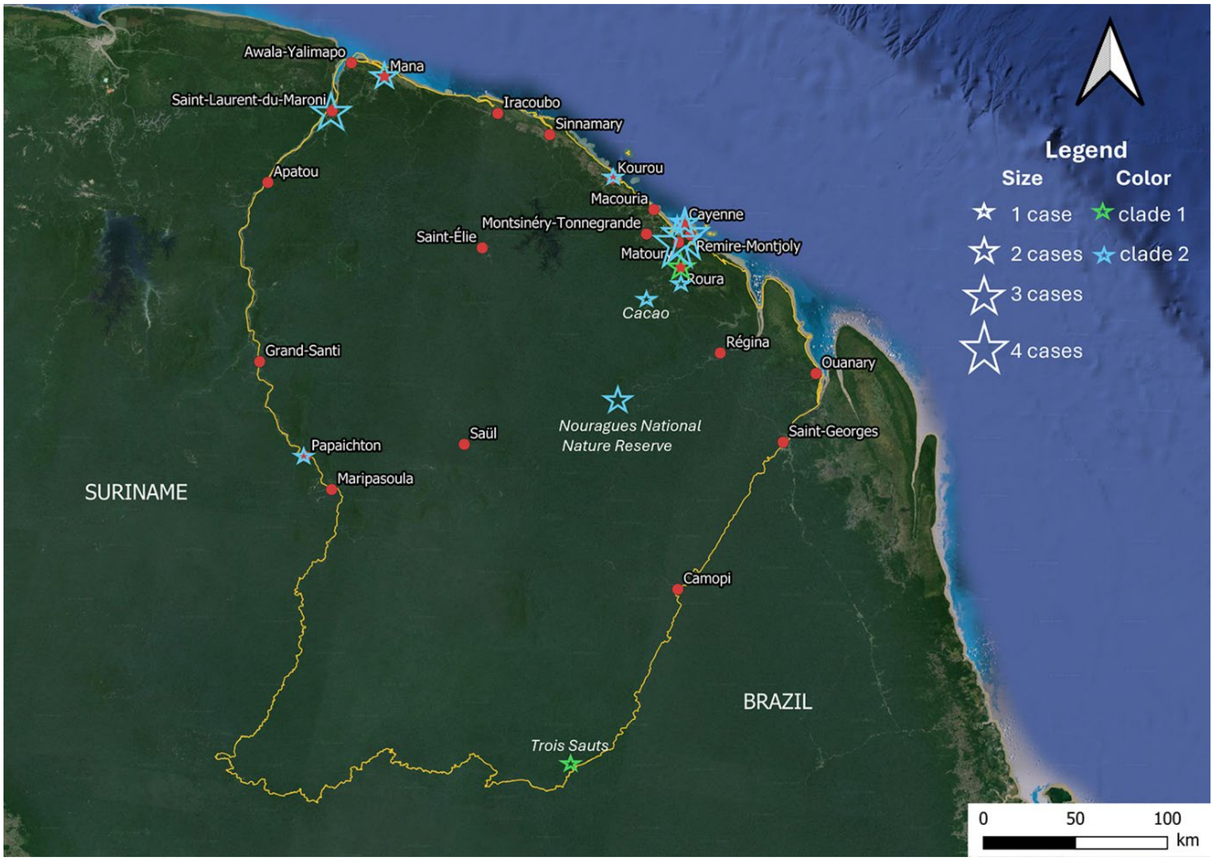

Appendix Figure. Map of French Guiana showing municipalities with communities or locations where patients are presumed to be infected, marked with a green star (sublineage 2, clade 1) or blue star (sublineage 2, clade 2).
